# Retraction: Acupuncture and nutritional parallels in obesity: a narrative review of multi-pathway modulation of the microbiota–gut–brain axis

**DOI:** 10.3389/fnut.2026.1894651

**Published:** 2026-06-02

**Authors:** Frontiers Editorial Office

The journal retracts the 07 August 2025 article cited above.

Following publication, concerns were raised regarding the citations included and scientific validity of the article. An investigation was conducted in accordance with Frontiers' policies. The authors failed to provide a satisfactory explanation and as a result, the conclusions of the article have been deemed unreliable, and the article has been retracted.

This retraction was approved by the Chief Editors of Frontiers in Nutrition and the Chief Executive Editor of Frontiers. The authors have not responded to correspondence regarding this retraction.

